# An Overview of Rhodoliths: Ecological Importance and Conservation Emergency

**DOI:** 10.3390/life13071556

**Published:** 2023-07-13

**Authors:** Dimítri de Araújo Costa, Marina Dolbeth, Martin Lindsey Christoffersen, Pamela Tatiana Zúñiga-Upegui, Márcia Venâncio, Reinaldo Farias Paiva de Lucena

**Affiliations:** 1 CIIMAR—Interdisciplinary Centre of Marine and Environmental Research, University of Porto, Terminal de Cruzeiros do Porto de Leixões, Av. General Norton de Matos, s/n, 4450-208 Matosinhos, Portugal; mdolbeth@ciimar.up.pt (M.D.); mvenancio@ciimar.up.pt (M.V.); 2 DSE—Department of Systematics and Ecology, CCEN—Center of Exact and Nature Sciences, UFPB—Federal University of Paraíba—Campus I, Cidade Universitária, João Pessoa 58050-585, Paraíba, Brazil; mlchrist@dse.ufpb.br (M.L.C.); rfplnal@gmail.com (R.F.P.d.L.); 3 ES-Inst—Environmental Smoke Institute, Rua Comerciante Antonio de Souza Lima, 25, Bairro Mangabeira, João Pessoa 58055-060, Paraíba, Brazil; 4 GIZ—Grupo de Investigación en Zoología, Facultad de Ciencias, UT—Universidad del Tolima, Barrio Santa Helena Parte Alta Cl 42 1-02, Ibagué 730006299, Colombia; pamelatutaina@gmail.com

**Keywords:** Corallinales, rhodolith beds, bioengineers, algae reefs, climate change

## Abstract

Red calcareous algae create bio-aggregations ecosystems constituted by carbonate calcium, with two main morphotypes: geniculate and non-geniculate structures (rhodoliths may form bio-encrustations on hard substrata or unattached nodules). This study presents a bibliographic review of the order Corallinales (specifically, rhodoliths), highlighting on morphology, ecology, diversity, related organisms, major anthropogenic influences on climate change and current conservation initiatives. These habitats are often widespread geographically and bathymetrically, occurring in the photic zone from the intertidal area to depths of 270 m. Due to its diverse morphology, this group offers a special biogenic environment that is favourable to epiphyte algae and a number of marine invertebrates. They also include holobiont microbiota made up of tiny eukaryotes, bacteria and viruses. The morphology of red calcareous algae and outside environmental conditions are thought to be the key forces regulating faunistic communities in algae reefs. The impacts of climate change, particularly those related to acidification, might substantially jeopardise the survival of the Corallinales. Despite the significance of these ecosystems, there are a number of anthropogenic stresses on them. Since there have been few attempts to conserve them, programs aimed at their conservation and management need to closely monitor their habitats, research the communities they are linked with and assess the effects they have on the environment.

## 1. Introduction

**Figure 1:**
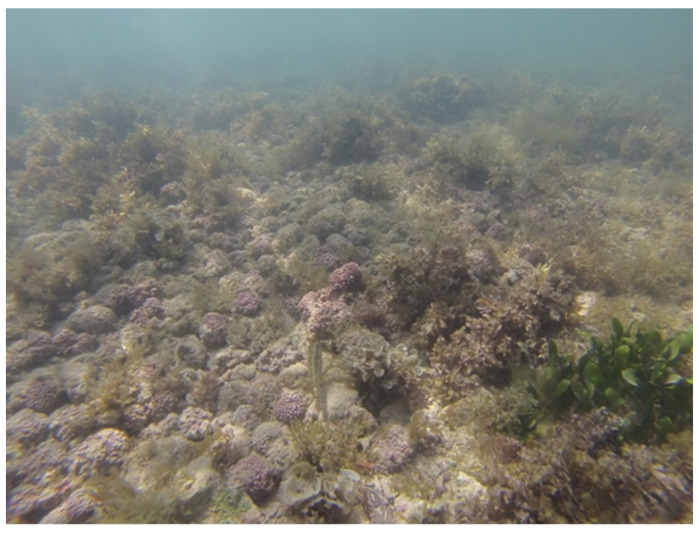
Rhodolith beds (remarkably, unattached nodule aggregation morphotypes) at the coast of the state of Paraíba, northeast Brazil. Photo: Massei, K.

**Figure 2:**
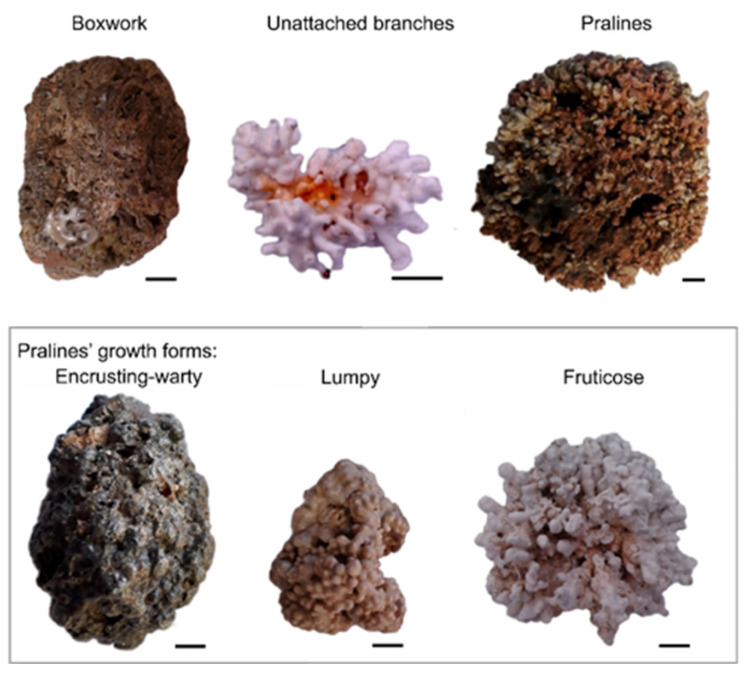
Rhodoliths’ morphology and pralines’ growth forms, collected along the shore of the Brazilian northeast state of Paraíba; scale: 1 cm. Photos: Costa, D.A.; Pereira, A.R.; Silva, F.A.; Silva, M.P.

Red calcareous algae create bio-aggregation ecosystems constituted by calcium carbonate (CaCO_3_), with two main morphotypes: (i) geniculate forms (i.e., articulate, “with uncalcified flexible genicula alternating with rigid calcareous inter-genicula” [1]) and (ii) non-geniculate structures [2,3,4], including bio-encrustations (CCA—crustose coralline algae) on hard-bottom substrata, such as rocks, boulders and pebbles, as well as unattached nodule aggregation morphotypes [5,6], designated here as “rhodoliths *stricto sensu*” (Figure 1 and Figure 2). When coralline red algae make up more than 50% of the volume of a biogenic nodule, the nodule is referred to as a “rhodolith”. The biogenic structure is otherwise referred to as a “coating”. [7,8,9]. As these algae reefs have calcium carbonate in their structure, similar to corals [10], they are important sinks for this chemical substance [11].

“Maërl” is another term for these biogenic formations, both alive and dead, that originated in Brittany (on the northwest coast of France) [5,12,13]. The term rhodolith is more prevalent worldwide, and the name “maërl” is mostly employed in research near the European Atlantic coast [7,14,15]. Rhodoliths are also known as “algaliths”, which are nodules the size of pebbles or non-articulate granules (also known as “oncoids”) produced from algae that exude carbonate [16]. Over time, several other names have also been employed, including numerous colloquial and scientific expressions. [17]. These different designations have contributed to hampering their definition, such as “boxwork rhodoliths”, “coatings”, “nucleated rhodoliths”, “gravels”, “nodules”, “rhodolites/rhodoliths”, “prâlines”, “algal balls”, “marls/maërls”, “coralline algal nodules”, “oncoliths/onkoids”, “rhodoids”, “algal-encrusted grains”, “rubbles”, “unattached branches” and “glagla” [9,18,19,20].

Although rhodoliths and maërl are quite similar in semantic terminology [21,22,23], rhodoliths include dead and alive unattached coralline red algae, whereas maërl only represents living, branched coralline thalli [24,25]. When its palaeoecological relevance was recognized, the word “rhodolith” was coined to describe a particular form of red stone [14,26]. Rhodoliths have become trustworthy markers in paleoenvironmental and palaeoecological surveys due to their low growth rate (between 0.01 and 5.0 mm per year) and carbonate nature, as well as their good preservation in fossil deposits [27,28,29,30,31,32,33,34]. These coralline algae have been extant across the earth for at least 55 million years, according to fossil evidence [35].

Rhodoliths are biogenic aggregations, which can have organic or inorganic nuclei that are either monospecific or multispecific in terms of the coralline algae they are composed of. In fact, it is possible that a number of non-articulate species are to blame for the extensive rhodolith beds that have formed on the ocean floor [17,36,37]. Other organisms may occasionally play an equal or even greater role as builders than coralline algae. For instance, benthic Foraminifera and coralline algae work together to generate “for-algaliths” [38,39]. The tubes of serpulid polychaetes may also be embedded into the nodules by the laminar thalli of coralline algae [38,40].

## 2. Geographical Distribution

One of the taxonomic groups with a wider geographic distribution range and larger climatic and bathymetric amplitudes include the rhodolith-forming algae [41]. The oceanic photic area is home to both living and dead calcareous structures, which are found from the intertidal zone to depths of 270 m [5,7,17,42]. Rhodoliths have been deemed suitable for studies on thermogeography because coralline algae undergo a mechanism known as “thermal control”, which causes their growth and reproduction rates to rise in warmer water [43].

The rhodolith beds are distributed from the Gulf of Alaska (Prince William Sound) to the Gulf of Mexico in North and Central America, [5,7,44,45] at depths of 50–90 m on the continental shelf [46]. These algae are also found along the Pacific coast of the southern hemisphere, from the Gulf of California to the South of Chile [47]. Rhodolith beds are widespread along the southern hemisphere’s Atlantic coast. On the Brazilian coast, for example, rhodolith beds may be the largest CaCO_3_ depositional area in the world (approximately 2 × 10^11^ tons) [48,49], particularly in the Abrolhos Bank region in northeast Brazil [4,48]. Additionally, rhodolith beds were found in the coral reef systems off the mouth of the Amazon River, covering an area of one thousand kilometres in length and 50 km in width, from French Guiana to the State of Maranhão (Brazil) [50].

On the European continent, these algae may be found on the Atlantic coastline—in passageways scoured by the tides and in protected regions with light currents, from the intertidal to 60 m depth—including Iceland, the Norwegian Sea (in deepest waters), the North Sea, the Baltic Sea, France, the United Kingdom, the Iberian Peninsula and the Canary Islands [44,51,52]—constituting a remarkably cold-adapted species [3], with a predominance of CCA forms (while Canary/Macaronesian rhodoliths are predominantly unattached forms/rhodoliths *stricto sensu*). Furthermore, along the Mediterranean coasts [51,53,54], e.g., in the Aegean Sea, Sardinia (Italy) and in Corsica and Marseille (France) [55], rhodolith-forming species are mainly represented by warm water-adapted and unattached red calcareous algae, which accumulate on the sea floor, from 9 to 150 m depths [3]. On this continent, Scotland features the most widespread rhodolith beds known, with 8000-year-old fossil records [56].

In Africa, rhodolith beds are widespread in the Gulf of Guinea, South Africa (such as on Príncipe’s island), and they can be found at depths of 5 m to 30 m [18,57,58]. In the Gulf of Aden, Northeastern Ethiopia [59] and in the Cape Verde islands [60], fossil rhodolith formations and sedimentary features that provide evidence for this habitat in the Pleistocene interglacial can be found.

Few of these non-geniculate red algae have been reported in Asia, from India to Russia [61,62,63,64]. Rhodolith beds, both alive and dead, are primarily found in Japan [65].

Finally, in Oceania, on the Great Australian Bight shaved shelves, rhodolith beds form a stable substrate conditions [66], being the primary successional coral-reef building stage for damaged reefs [67,68,69]. Rhodoliths can also be found in New Zealand, e.g., around the North Island [70]. These rhodolith beds are approximately 6500 years old [71], and are integral parts of the processes of building, erosion, burial, and recolonization [72].

Regarding algae species composition, the order Corallinales Silva & Johansen, 1986, represents the largest group in Rhodophyta, with around 840 species [73,74,75], classified systematically on the AlgaeBase website [74].

## 3. Rhodoliths’ General Morphological Characteristics

The rhodoliths’ morphology and phytal composition (i.e., related fauna in algae) and, consequently, the heterogeneity of rhodolith beds, are strongly influenced by hydrodynamism and light [76]. Rhodoliths are usually more common in zones with moderate to high-energy currents (excluding the *L. corallioides* and *P. calcareum* mentioned above) [21,77]. However, they may exhibit significant morphological polymorphism, which is associated with hydrodynamism: the size, shape and the pattern of the ramification of the rhodoliths are mostly influenced by local hydrodynamics [7,36]. The species composition of rhodolith beds, on the other hand, is also influenced by light penetration and depth, sedimentation, the temperature of the water, salinity and water quality [21], among other factors. This complexity of conditions commonly causes sporadic and irregular distribution patterns of rhodoliths on the ocean floor [36], where the rhodolith beds intercalate with sandy bottoms, forming mosaics within marine environments [78].

Rhodoliths can be divided into three types based on their morphology: boxwork, unattached branches and pralines (Figure 2, e.g., [1,8,41,79]):

(a) Boxwork: multi-specific and irregular nodules with inside spaces filled by sediment and a core containing a small pebble or biogenic remnant;
(b) Unattached branches: rhodoliths lacking a macroscopic nucleus and potentially characterized by a high degree of protuberance;
(c) Pralines: compact mono (oligo)specific nodules, with a lytic or biogenic nucleus, with protuberances that have developed strongly on the surface.

The growth pattern of rhodoliths of the pralines type can be used for further classification (Figure 2, [8,80,81]):

(a) Warty growth: branches can be cylindrical or compact, and are typically organized radially;
(b) Lumpy growth: protuberances are enlarged, numerous and contiguous and rarely branched;
(c) Fruticose growth: branches are remarkably detached from each other.

Other species, both fauna and flora, are provided with shelter and safety by the complexity formed by the accumulation of the rhodolith nodules of various shapes (i.e., spherical and elliptical shapes of distinct sizes, ranging from a few millimetres to several centimetres). The associated flora also increases the complexity of the rhodolith, which serves as a micro-ecosystem [82] and may contain different fauna. This micro-ecosystem may be fundamentally dependent on indirect positive interactions (such as mutualism and commensalism), which involve successions of positive interspecific interactions, a phenomenon known as “facilitation cascades” [83]. This process is applied in hierarchically organized communities where a basal habitat-former (characteristically a large primary producer, e.g., the rhodolith) creates living space for an intermediate habitat-former (e.g., an algal epiphyte), which in turn produces living space for the focal organisms (e.g., polychaetes, echinoderms, molluscs and crustaceans).

## 4. Associated Biota

Rhodoliths are bioengineers (“biological builders” or “ecosystem engineers”), that supply an irregularly shaped three-dimensional environment [76,84] and provide food for multiple marine organisms [4]. Due to the variety of niches it offers, the rhodolith provides shelter and protection for associated fauna against physical disturbances and predators and allows coexistence [75,85]. Rhodoliths have been recognised as places of the recruitment of marine species, providing refuge for the early stages of growth of commercially significant fishes [17] and for several species of marine invertebrates [17,82,86,87,88,89], such as polychaetes, bivalves, molluscs and echinoderms [81,85,90,91,92]. A rhodolith’s internal space may further contain particles of organic matter for filtering organisms and may provide microphytobenthos for herbivorous scavengers [93].

The rhodolith’s inhabiting organisms can be divided into endobionts and ectobionts. The endobiota inhabit the interior of the rhodoliths, being represented mostly by the following taxonomic groups: Mollusca, Echinodermata and Polychaeta. The ectobiota living on its surface belong primarily to cnidaria, crustacea and lithophytic/epilithic algae.

The composition of macroalgal assemblages on rhodolith beds can vary greatly, depending on the habitat and the depth at which the bed develops. The associated macroalgal assemblages are normally characterized by sciaphilous and reophilous species in deeper beds (e.g., *Osmundaria* spp., *Sargassum* spp., *Sporochnus* spp. and *Phyllariopsis* spp.) and by more generalist species in shallower beds (such as the genera *Corallina* spp., *Gelidium* spp., *Gracilaria* spp., *Caulerpa* spp., *Codium* spp., *Halimeda* spp., *Dictyota* spp. and *Padina* spp.) [94,95,96,97].

The key factors controlling the faunistic communities connected to rhodolith beds are both the morphological characteristics of the rhodolith and the environmental characteristics in which they are found [98,99]. Usually, the abundance and diversity of the associated fauna may increase with the size and heterogeneity of the rhodolith [22,82,100,101]. Yet, studies confirming this hypothesis are still incipient [102].

### Functional Approach

Regarding the functional composition of the associated fauna with rhodoliths, few studies have attempted to understand their dynamics and how the external environment may affect them. However, information on functional diversity can lead to a better understanding of the ecological trade-offs and roles of the species within the ecosystem [103], particularly when considering the rhodolith as a micro-ecosystem. Most studies tackling functional aspects have focused on feeding or trophic guilds and interactions [12].

Still, a more comprehensive approach may provide a deeper understanding of the interplay between rhodoliths and their associated fauna [90]. For instance, a recent study from northeast Brazil (with 144 analysed rhodoliths), combining different traits for the associated invertebrates’ communities, found differences in their functional identity when comparing three beaches with different anthropogenic impacts [90]. Most invertebrates living on the rhodoliths of less polluted beaches were polychaetes and echinoderms that were predators and biodiffusors with slow free movement and high fecundity [90]. These characteristics appear to be beneficial for a three-dimensional environment like rhodoliths, fostering food, habitat and protection for the resident fauna, while the fauna might benefit from the rhodolith itself via oxygenation and sediment remobilization. However, the invertebrate community from the most contaminated beach had distinctive traits, including a higher occurrence of suspended feeder bivalves with restricted mobility and the absence of several functions seen on the two better preserved beaches, which may be crucial for the survival of rhodolith habitats [90].

## 5. Ecological Importance as Regulation Services

Besides the fundamental role of rhodolith beds as habitat for a variety of biota, as detailed above, from an environmental perspective, rhodolith beds serve an essential function in maintaining marine pH levels [28,104], due to the high concentrations of carbonates that accumulate in their structures.

Rhodoliths also play a crucial role for climate regulation processes by intervening in the global cycles of diverse chemical elements [4]. An example is the biogenic gas dimethylsulphide (DMS), which plays “an important role in the Earth’s albedo, and thus climate regulation, through the formation of aerosols and cloud condensation nuclei” [105]. Recent studies have also highlighted their potential as blue carbon by sequestering atmospheric CO_2_ and storing it for millennia [106,107]. This process of carbon storage may occur within the rhodolith tissue and through the sediment with organic material trapped within the bed, yet the stability of these stores are still being assessed [107].

Additionally, the production of dissolved organic carbon and calcium carbonate promotes the proliferation of other organisms within the ecosystem [107]. When associated with rhodoliths, a group of organisms formed by bacteria, viruses and small eukaryotes acts as an ecological unit and is designated as holobiont [108], whose function is intimately related to the associated microbiome [109]. This symbiotic microbiota may react quickly to changes in the ambient conditions, offering an effective mechanism for acclimatization through mechanisms of adaptation (i.e., phenotypic plasticity). For instance, under the conditions of increased carbon dioxide partial pressure (*p*CO_2_) and seawater temperature, specific host-associated microorganisms may experience changes in abundance, altering the fitness and function of the holobiont [110]. However, it is still unknown how certain climate change factors, such as ocean acidification, may affect the holobiont community [109]. Additionally, due to the importance of calcium carbonate substrates in the life cycle of many macroalgae, this community (microorganisms and rhodoliths) is crucial for the maintenance of marine ecosystems [46].

Generally, the ecological services offered by rhodoliths vary depending on the geographic area. In tropical areas, these algae serve as calcified substrates for other organisms [111]. Additionally, they can have a physical stabilizing effect that facilitates coral settlement and the establishment of coral reefs across geologic timescales [112]. Water filtration and pH regulation in waters and sediments, as well as additives for animal food (raw material production), are among the described ecosystem services related to the rhodoliths beds [21,113].

## 6. Climate Change and Anthropogenic Impacts

The reduction in marine biodiversity, including calcareous organisms (such as coral reefs and rhodoliths beds), is ultimately caused by ocean acidification and global warming and their associated effects, such as changes in carbon chemistry and sea level rise, leading to changes in the way the marine ecosystem functions [75,114,115,116]. These algae’s capacity for carbon sequestration (the absorption and storage of atmospheric CO_2_) may be equivalent to the coral reefs in extent and productivity [48], so that rhodolith have been considered “algae reefs” [35]. The calcification of the rhodoliths, however, may be negatively impacted by ocean acidification through the increase in *p*CO_2_ levels due to the high dissolubility of their calcite and magnesium structures [117], the phenomenon known as “coralline algae bleaching”, which results in the depletion of photosynthetic pigments and may lead to necrosis [118]. Nevertheless, dissolved inorganic carbon may favour the growth of foliose algae (“fleshy algae”) to the detriment of calcareous algae [117,119].

Additionally, the levels of dissolved inorganic carbon, alkalinity and oxygen, besides affecting the calcification rates of the coralline algae, may also affect photosynthesis and respiration rates [117], whose effects may be intensified by the increase in temperature, as documented, e.g., for *Porolithon onkodes* (Heydrich) Foslie 1909 from Australia [120] and *Lithophyllum cabiochae* (Boudouresque & Verlaque) Athanasiadis 1999 from the Mediterranean Sea (France) [121].

Still, the effects of the climate conditions could be very species-specific. Some species, such as *Bossiella orbigniana* (Decaisne) Silva 1957, *Neogoniolithon* sp., *Lithothamnion glaciale* and *Lithophyllum cabiochae*, have the ability to maintain high internal pH to precipitate CaCO_3_, despite the reduction in environmental pH [122,123,124]. But as a result, these species would be more susceptible to pathogens [125]. Additionally, these negative effects could result in a decline in photosynthetic activity, compromising the algae metabolism, ultimately resulting in the death of rhodoliths and the associated fauna [126].

**Figure 3:**
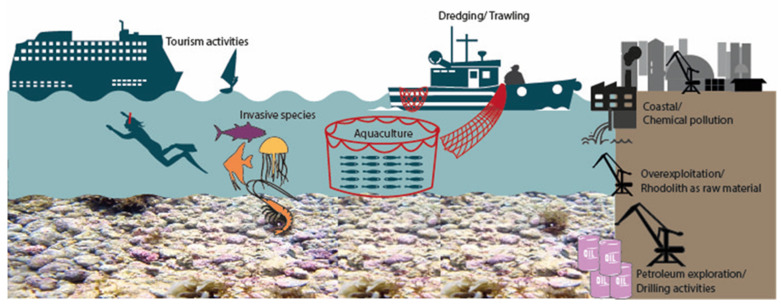
Schematic representation of main anthropogenic factors affecting the rhodolith beds. Figure produced using Canva software (https://www.canva.com/, accessed on 15 February 2023), with a Creative Commons (CC BY 4.0) license for images/drawings. Photo (rhodolith beds): Massei, K.

In addition to the climate impacts, rhodolith beds have low resilience and, consequently, they are extremely susceptible to anthropogenic activities, such as dredging/trawling, aquaculture, coastal chemical pollution, petroleum exploration/drilling activities, tourism activities, overexploitation, and the indirect effects of invasive species [95,127,128]. These anthropogenic activities and their potential effects on rhodolith beds are summarized in Table 1 and Figure 3.

### Environmental Stressors

CaCO_3_-ionized availability in marine water is essential for rhodolith-forming red algae species to build their three-dimensional carbonate structures via assimilation process/carbon sequestration, which is dependent on the ocean balance [4]. In this way, rhodolith beds are very sensitive to variations in chemical–physical parameters, which in turn affect their growth and survival. As example, pH decreases linked to the release of pollutant gas is the main factor (“our emphasis”) that drives the “health” of the beds.

Hydrodynamic forces correspond a fundamental driver for distribution and shape-delineation of the rhodolith beds through a phenomenon that relocates and creates the unattached nodules. Wave activity, intensified by storms, promotes significant rhodolith beds mobility and morphology-formation [137].

Many rhodolith-forming red algae species are adapted to water temperature conditions that dictate the occurrence of colder and warmer taxa worldwide. In temperate areas, rhodolith beds are abundant, particularly in Europe, where the species *Lithothamnion glaciale* Kjellman, 1883; *Lithothamnion corallioides* (Crouan & Crouan) Crouan & Crouan, 1867; *Lithothamnion tophiforme* (Esper) Unger 1858; and *Phymatolithon calcareum* (Pallas) Adey & McKibbin ex Woelkering & Irvine, 1986, dominate [12,21,44,53]. The similar ecological niches of *L. corallioides* and *P. calcareum* are influenced by environmental drivers, such as wave motion and seabed currents—resulting from rivers, tides and salinity differentials—and the interaction between depth, light accessibility and water quality in general [21,41,138,139]. These coralline algae are typically better adapted to low-energy oceanographic environments (they usually do not occur when exposed to strong wave activity), more frequently found in coves and bays [21].

Water temperature also has a significant impact on the species composition of rhodoliths, principally in the North Atlantic [140]. For example, the absence of *L. corallioides* in Scotland has been attributed either to the wintertime low temperatures not allowing the survival of this species (ideal value between 2 and 5 °C) or to the lack of high temperatures lasting long enough to support the species’ annual growth [21]. On the other hand, *L. glaciale* appears to be restricted to areas on the north of the British Isles [21], requiring low temperatures for reproduction (<9 °C, e.g., [141]).

Indeed, ocean warming can trigger tropicalization events [138], which are characterized by the gradual replacement of cold-adapted rhodoliths with warm-adapted ones.

## 7. Rhodolith Bed Preservation and Protection Initiatives

Rhodoliths are a unique biotope in the world, yet despite this, they are constantly endangered due to climate change and anthropogenic pressures (as stated above). Additionally, there are insufficient protection measures and a general lack of understanding of how these structures function as an ecosystem [17]. Nevertheless, to prevent the extinction of such unique and significant environments, considered an unrenewable resource, various conservation and protection programs are expanding globally [142]. Examples of such protection measures are the habitat directives of the European Commission, i.e., Directive n° 82/72/EEC—Bern Convention [143]; 92/43/EEC—‘Natura 2000’ [2,144], category 1170: reefs [17,145]; and Council Regulation 1967/2006 [146]. All 28 countries of the European Union are covered by Natura 2000, which has authority over both land and sea [145].

The goal of these habitat directives is to ensure both the maintenance of conservation status and its expansion, and the rhodolith-forming algae species *L. corallioides* and *P. calcareum* are species included in priority habitats [144]. In Europe, there is also the ‘Biomaërl’ programme, which was started in 1996 in the United Kingdom, France, Spain and Malta, the primary goals of which are to protect and monitor biodiversity related to the maërl beds in the NE Atlantic and Mediterranean, as well as to evaluate principal anthropogenic impacts [53].

Only a few protected marine areas in the Eastern Pacific consider rhodolith-forming algae as important habitats for preservation, e.g., in Mexico [23] and in isolated areas in Costa Rica and Panama [147].

In Asia, there are certain UNESCO global geoparks that comprise rhodolith banks, e.g., Shimabara Peninsula in Kyushu, Japan (Unzen Volcanic Geopark), the Jeju Island in Korea (Jeju Island Geopark) [148] and certain local parks in Japan [149].

“ReBentos” (“Rede de Monitoramento de Habitats Bentônicos Costeiros”) is a marine monitoring network in Brazil that aims to establish an integrated network of studies on benthic habitats along the Brazilian coastline, including vegetated submerged sea floors, rocky shores, beaches, marshes, mangroves, coral reefs, rhodolith benches and estuaries, all integrated with environmental education activities [150]. ReBentos is linked to the National Institute of Science and Technology for Climatic Changes (“Instituto Nacional de Ciência e Tecnologia para Mudanças Climáticas”, INCT-MC) and to the “Coastal Zones Subnet from CLIMATE Network” (Brazilian Ministry of Science, Technology, Innovation and Communications). This organization’s mission is to provide scientific bases for identifying the effects of regional and global environmental changes in the stated benthic habitats, including rhodolith beds, and to serve as vital tool for the implementation of effective environmental management strategies. This organisation has thus begun the collection of historical data of marine biodiversity along the Brazilian coast [150,151]. Nevertheless, the studies are still ongoing, with no results published so far.

Moreover, in the context of conservation, environmental education can be a crucial technique for raising local understanding among those who are enjoying these marine communities [152], including the rhodolith beds and associated biota [153,154]. In this sense, safeguarding coastal regions could benefit the tourist sector by preventing deterioration through visitors in such zones [136].

## 8. Conclusions

Rhodolith beds, composed of non-geniculate red calcareous algae, are essential marine habitats due to their remarkable ability to assimilate calcium carbonate and to shelter resident biota. The main taxon that makes up calcareous algae is the order Corallinales, which is found in all oceans, mainly in tropical and temperate zones. The habitat is known for variety of ecosystem services, such as steaming from nursery areas, as a habitat for a variety of species and sediment stabilization, as well as their important role in balancing biogeochemical cycles, including blue carbon potential and stabilizing pH. Nonetheless, this calcareous habitat is sensitive to a variety of human activities, such as dredging, pollution, aquaculture, tourism effects and, ultimately, climate change, from which ocean acidification is among the major stressors compromising CaCO_3_ assimilation. Thus, it is crucial to continue conducting studies on rhodolith beds to gain a better understanding of their ecosystem service potential, including blue carbon; to adopt functional diversity in order to understand the dynamics of rhodolith-associated fauna; and to develop more comprehensive protection actions schemes to ensure their conservation.

## Figures and Tables

**Table 1:** Examples of consequences from the main anthropogenic activities affecting rhodoliths beds.

Drivers	Examples of Consequences/Impacts	References
Dredging/trawling	Production of a plume of fine sediment that amplifies the effect on the remaining organisms; physical perturbation in the fauna and associated algae; release of blue carbon stored in the beds	[129,130,131]
Coastal chemical pollution	Impacts the carbon cycle and algae’s ability to sequester carbonate	[117]
Aquaculture (including fish farming)	Due to deposition of fine sediments with high organic matter content/waste dispersion, the associated fauna changes, decreasing their complexity and biodiversity	[26,132,133]
Petroleum exploration/drilling activities	Disturbs photosynthesis due to sediment suspension; induces the burial of the algae	[134,135]
Overexploitation—rhodolith as raw material	Declines rhodoliths and associated fauna	[134]
Tourism activities	Alterations in the associated fauna due to increased and seasonal organic pollution; the trampling of the sediment beds containing rhodoliths	[136]
Indirect effects of invasive species/ecological competition	Excrement from invasive molluscs (e.g., *Crepidula fornicuta*) cover the spaces between the stalks of the rhodoliths or the ocean floor is covered by fleshy and/or invasive algae that make rhodoliths more vulnerable [95]	[12,44]

## Data Availability

This study did not report any data that needs to be available.
